# Two glyoxylate reductase isoforms are functionally redundant but required under high photorespiration conditions in rice

**DOI:** 10.1186/s12870-020-02568-0

**Published:** 2020-07-29

**Authors:** Zhisheng Zhang, Xiu Liang, Lei Lu, Zheng Xu, Jiayu Huang, Han He, Xinxiang Peng

**Affiliations:** 1 State Key Laboratory for Conservation and Utilization of Subtropical Agro-bioresources, College of Life Sciences, South China Agricultural University, Guangzhou, China; 2 Guangdong Laboratory for Lingnan Modern Agricultural Science and Technology, South China Agricultural University, Guangzhou, China; 3 Institute of Fruit Tree Research, Guangdong Academy of Agricultural Sciences, Guangzhou, China

**Keywords:** Glyoxylate reductase, Glyoxylate, Photorespiration, Oxalate, Rice

## Abstract

**Background:**

The glyoxylate reductase (GR) multigene family has been described in various plant species, their isoforms show different biochemical features in plants. However, few studies have addressed the biological roles of GR isozymes, especially for rice.

**Results:**

Here, we report a detailed analysis of the enzymatic properties and physiological roles of OsGR1 and OsGR2 in rice. The results showed that both enzymes prefer NADPH to NADH as cofactor, and the NADPH-dependent glyoxylate reducing activity represents the major GR activity in various tissues and at different growth stages; and OsGR1 proteins were more abundant than OsGR2, which is also a major contributor to total GR activities. By generating and characterizing various *OsGR*-genetically modified rice lines, including overexpression, single and double-knockout lines, we found that no phenotypic differences occur among the various transgenic lines under normal growth conditions, while a dwarfish growth phenotype was noticed under photorespiration-promoted conditions.

**Conclusion:**

Our results suggest that OsGR1 and OsGR2, with distinct enzymatic characteristics, function redundantly in detoxifying glyoxylate in rice plants under normal growth conditions, whereas both are simultaneously required under high photorespiration conditions.

## Background

Plant glyoxylate reductase (GR; EC 1.1.1.26/79) is a key enzyme involved in aldehydes metabolism, which catalyzes the reduction of glyoxylate and succinic semialdehyde (SSA) to glycolate and γ-hydroxybutyrate using NAD(P) H as a cofactor [1, 2]. GR activity was first detected in crude extracts of spinach leaves. Subsequently, GR was successfully purified from tobacco and spinach leaves; both of which prefer NADPH to NADH as cofactor [3–5]. Later on, two GR isozymes were molecularly identified in *Arabidopsis thaliana*, further analysis confirmed that AtGR1 (At3g25530) is localized to the cytosol while AtGR2 (At1g17650) is localized in plastids and mitochondria [6]. Recently, with NADPH as cofactor, the catalytic characteristics of GR isozymes from apple, rice and *A.thaliana* were analyzed, showing that glyoxylate (*K*_*m*_ = 19.1 ~ 53.2 μM) is preferred over SSA (*K*_*m*_ = 870 ~ 8960 μM) as substrate for all GR isoforms [6, 7].

Previous evidences found that expression of *GR* genes was upregulated under abiotic stresses (e.g. salinity, drought and submergence), accompanied by the accumulation of γ-hydroxybutyrate, suggesting that GR is involved in the detoxification of SSA in response to abiotic stresses [8, 9]. Zarei et al. [7] showed that in the presence of exogenous glyoxylate, *AtGR*-RNAi lines are more sensitive to chilling stress than wild type (WT), and characterization of *AtGR*-overexpression lines further support that glyoxylate reduction catalyzed by AtGRs is closely related to stress resistance of *A. thaliana*. It is noteworthy that a major source for glyoxylate production is photorespiratory metabolism in plants, where glycolate is oxidized to glyoxylate by glycolate oxidase (GLO) [10–12]. Generally, photorespiratory glyoxylate is quickly detoxified by serine:glyoxylate aminotransferase and glutamate:glyoxylate aminotransferase in peroxisomes, but it has been considered that the glyoxylate leaking from peroxisomes under photorespiration-promoting conditions needs to be scavenged by GRs [10, 11, 13]. However, no molecular genetic evidences have been presented so far to prove this opinion. In addition, glyoxylate is an efficient precursor for oxalate biosynthesis in plants [14], and various functions have been reported for oxalate, including heavy metal detoxification, ion balance, pathogen defense and tissue support [15, 16]. It has been noticed that oxalate regulation does not necessarily depends on photorespiratory rates [14, 17], which may be due to some other anaplerotic pathways that have participated in glyoxylate regulation [7, 14, 18, 19]. Hence, oxalate generation from glyoxylate is also likely to be regulated in a complex manner, and it has not yet been reported whether GR mediates the regulation of glyoxylate-dependent oxalate accumulation.

To date, *GR* genes were identified in various plant species [20], but only the GR isoforms from *A.thaliana* were molecularly and biochemically characterized [6, 20]. The function and mechanism of GRs are still largely unknown, and particularly few studies addressed in vivo roles of GRs in crop plants. By performing BLAST with the sequences of *AtGR1* and *AtGR2*, two *GR* homologs in the rice genome were identified (i.e. *OsGR1*-Os02g0562700 and *OsGR2*-Os01g0574600). Here, to understand the catalytic properties and physiological roles related to glyoxylate metabolism, OsGR1 and OsGR2 were biochemically and genetically investigated. The results showed that the two OsGR isozymes, localized in the cytosol or chloroplasts, had distinct biochemical and enzymatical properties. Further, we generated various genetically modified rice lines for both *OsGR* genes. There were no phenotypic differences noticed for single or double *OsGR* mutants under normal natural conditions and even no differences were observed for either single mutant under high photorespiration conditions, whereas a stunted growth was noticed only for the *OsGR1/OsGR2* double mutants under high photorespiration conditions. Taken together, our results demonstrate that the two OsGR isoforms, with distinct enzymatic characteristics, are functionally redundant but both are simultaneouly required under high photorespiration conditions in rice.

## Results

### Expression patterns and subcellular localization of OsGR1 and OsGR2

**Fig. 1:**
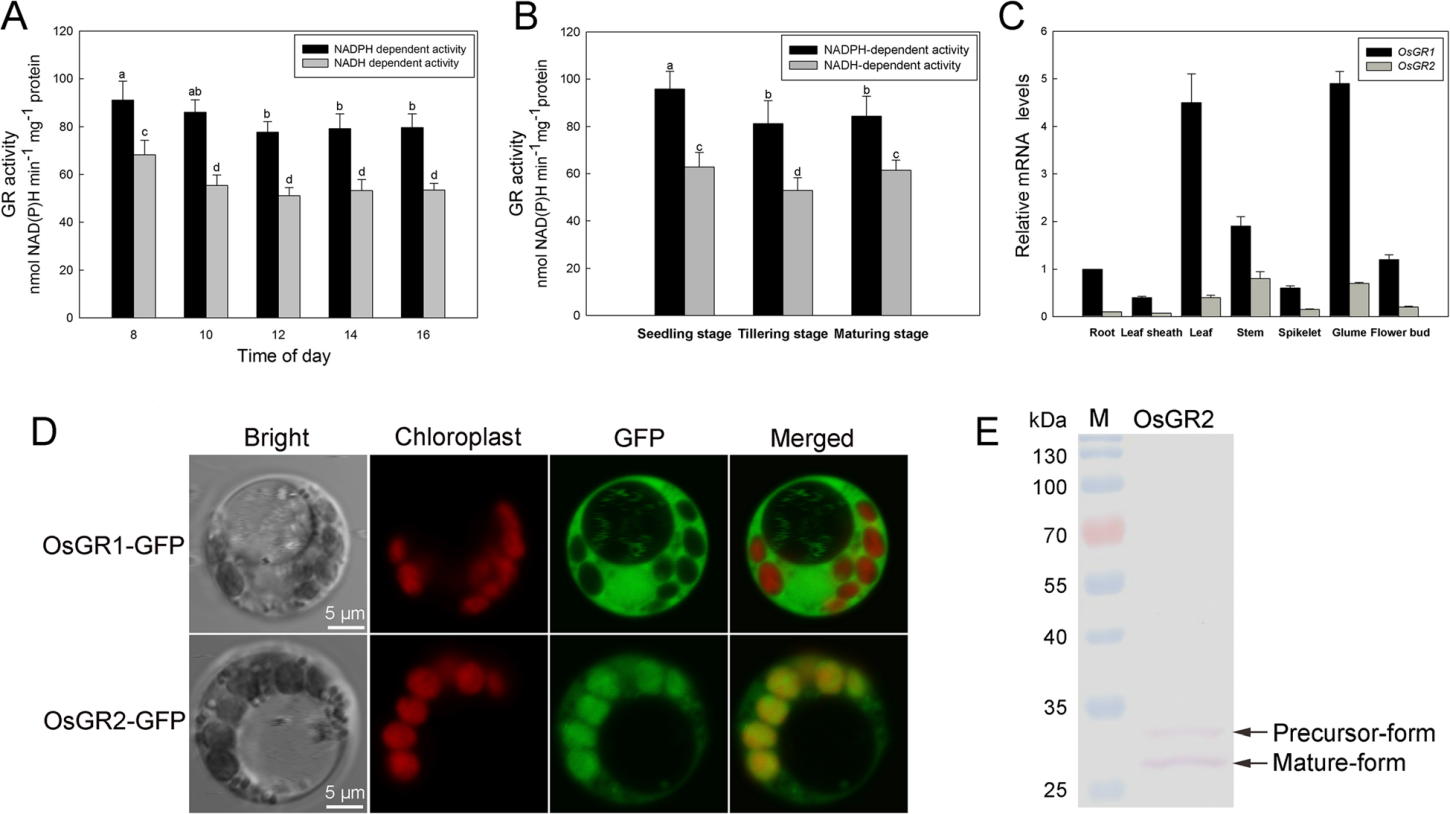
Expression patterns and subcellular localization of OsGR isoforms. **a** The diurnal changes of NAD(P)H-dependent GR activities in rice leaves. The second leaf from the top was detached from rice plants at the 5-leaf stage for determination. **b** The NAD(P)H-dependent GR activities at different developmental stages of rice plants. The second leaf from the top was detached for determination. **c** mRNA transcript abundances of *OsGR1* and *OsGR2* in different tissues were determined by qRT-PCR. Relative mRNA levels in various tissues were graphed based on the *OsGR1* mRNA level in root as 1. Values are means ± SD of three replicates. Means denoted by the same letter did not significantly differ at *P* < 0.05 according to Duncan’s multiple range tests. **d** Subcellular localization of OsGR1 and OsGR2. The OsGR-GFP fusion constructs were transiently expressed in rice protoplasts, cells are imaged by a confocal microscope at 14–16 h after the transfection. The red signals represent chlorophyll autofluorescence. The result is representative of three independent experiments. Scale bar, 5 μm. **e** Proteins extracted from the OsGR2-GFP transformed protoplasts were analyzed by western blot using a monoclonal anti-GFP antibody. Precursor-form is the precursor protein of OsGR2-GFP synthesized in cytosol with an N-terminal CTP, Mature-form is the OsGR2-GFP located in choloroplast and the N-terminal CTP is cleaved off. The result is representative of three independent experiments

Up to now, *AtGR1* and *AtGR2* are most extensively studied among all of the *GRs* from different plants [6, 8, 9]. The rice genome contains two *GR* homologs: *OsGR1* located on chromosome 2, and *OsGR2* seated on chromosome 1. The similarity between *OsGR1*, *OsGR2*, *AtGR1* and *AtGR2* is appreciably high (Table 1, Additional file 1). Both OsGR1 and OsGR2 may use NAD(P) H as cofactor, so we detected the total NAD(P)H-dependent GR activities in rice leaves separately. The NADPH-dependent activity was much higher than the NADH-dependent activity, both of which showed a fluctuation throughout the day (Fig. 1a). Besides, GR activities displayed variation during different growth stages, being highest at the vigorous vegetative stage (Fig. 1b). Transcript abundances of the two *OsGR* genes were determined by real-time quantitative PCR (qRT-PCR). As shown in Fig. 1c, *OsGR1* displayed much higher transcriptional levels than *OsGR2* in all organs tested. Moreover, *OsGR1* was abundant in leaves and glume, and moderately expressed in other organs, whereas *OsGR2* was expressed primarily in leaf sheaths, glume and leaves. Proteomic data suggested that the protein abundance ratio of OsGR1 to OsGR2 ranged from 1.2 to 1.4 in leaves (Table 2). The results indicate that both OsGR1 and OsGR2 are widely distributed in photoautotrophic tissues, and their NADPH-dependent activities contribute mostly to total GR activities in rice.

**Table 1:** Similarities of OsGR and AtGR isoforms at the level of protein and nucleotide

Protein	*OsGR1*	*OsGR2*	*AtGR1*	*AtGR2*
mRNA				
*OsGR1*		58.1%	80.7%	58.2%
*OsGR2*	61.7%		58.0%	72.7%
*AtGR1*	58.7%	49.3%		58.2%
*AtGR2*	58.4%	56.5%	45.6%	

Lipman-Pearson method was used for protein sequence alignment, Wilbur-Lipman method was used for mRNA sequence alignment

**Table 2:** Protein relative abundances of OsGR1 and OsGR2 in WT detected by proteomic analysis

Proteins	Acesssion	Coverage [%]	Unique Peptides	Abundances (Normalized) Sample 1	Abundances (Normalizd) Sample 2	Abundances (Normalizd) Sample 3
OSGR1	Q84VC8	57	13	668.1	648.5	691
OSGR2	Q656T5	46	9	561.3	475	511

Total proteins from three independent WT plants (Sample 1, 2 and 3) were used for proteomic analysis

Subcellular localization of OsGR1 and OsGR2 was first predicted by the WoLF PSORT and Plant-mPLoc [21, 22], turning out that OsGR1 could be a cytoplasmic protein and OsGR2 likely located in the chloroplast. To experimentally prove the prediction, OsGR1 and OsGR2 fused with green fluorescence protein (GFP) were transiently expressed in rice protoplasts. As shown in Fig. 1d, OsGR1-GFP was observed to localize in the cytosol, while OsGR2-GFP in the chloroplast. Generally, chloroplast proteins are synthesized in cytosol, with an N-terminal chloroplast transit peptide (CTP) linked as a precursor form; the precursor form is guided into chloroplast by the CTP, which is then proteolytically removed after import into the chloroplast [23, 24]. Proteins extracted from the rice protoplasts transiently expressing the OsGR2-GFP were used for western blot assay with anti-GFP antibody. The results showed that a large number of OsGR2-GFP proteins were detected as mature form without CTP (Fig. 1e), further proving that OsGR2 was localized in the chloroplast.

### Enzymatic characterization of OsGR1 and OsGR2

**Fig. 2:**
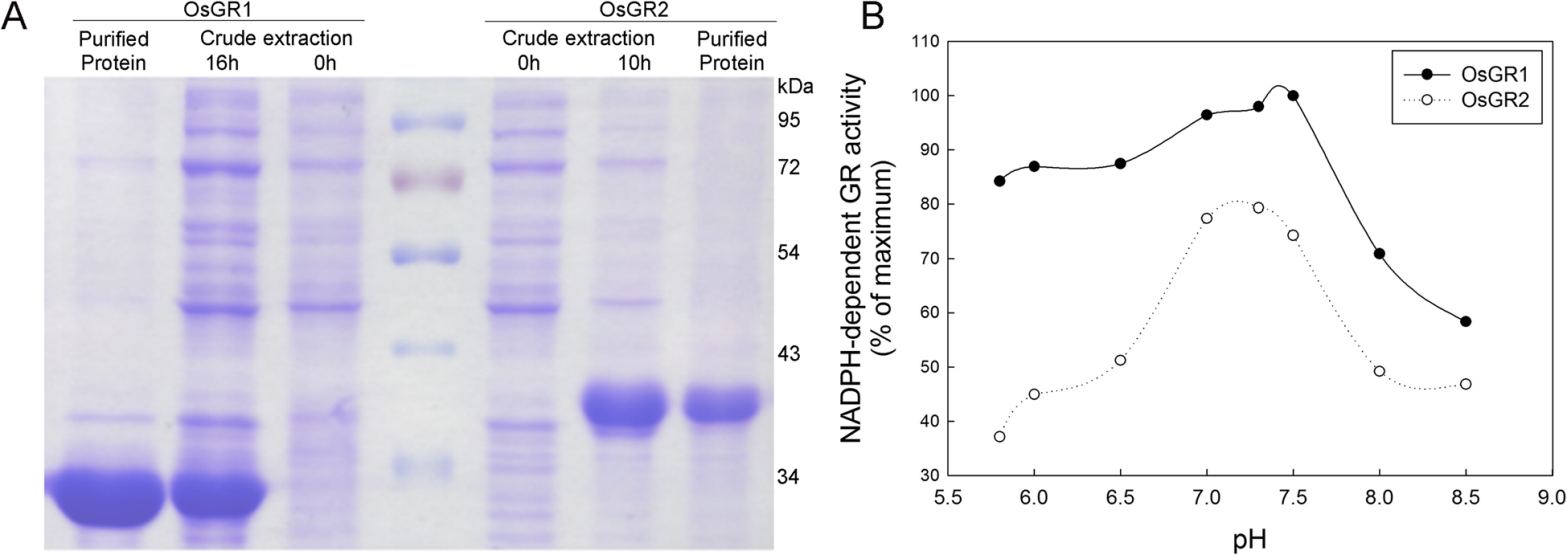
Expression of OsGR isoforms in *E.coli*. **a** OsGR1 and OsGR2 were expressed in *E.coli* cells and then purified using immobilized metal affinity chromatography, their purity and subunit molecular weights were determined by SDS-PAGE. The results are representative of three independent experiments. **b** Effect of pH on the NADPH-dependent activities of OsGR1 and OsGR2. The activities were detected at a physiological temperature (30 °C), and activities were expressed as a percentage of the maximum value, each point represents the mean of three determinations

There are few studies about enzymatic properties of the OsGR isozymes. A preliminary research has been recently made on NADPH kinetics of OsGR1 and OsGR2 [7]. To further explore functions of OsGR1 and OsGR2, we comparatively analyzed glyoxylate-dependent enzymatic differences of the two OsGR isoforms. A 6 × His-tag was fused to a full-length *OsGR1* and a truncated *OsGR2* sequence (without N-terminal CTP), then these *OsGR* sequences were cloned into pColdIV vector and expressed in *E.coli*. The OsGR1 and OsGR2 proteins were subsequently purified from the *E.coli* crude extract by His-tag affinity chromatography. SDS-PAGE analysis showed that molecular weights of OsGR1 and OsGR2 subunits were approximately 30 kDa and 37 kDa, respectively (Fig. 2a); similar optimum pH in the range of 7.0–7.5 was observed for OsGR1 and OsGR2 (Fig. 2b). The kinetic analyses were performed at physiological temperature (30 °C) and pH 7.4. As shown in Table 3, OsGR1 and OsGR2 exhibited a much higher affinity for NADPH than for NADH. The glyoxylate-linked *K*_*m*(NADPH)_ values of OsGR1 and OsGR2 were 17.6 and 53 μM, while the *K*_*m*(NADH)_ values were 420.2 and 403.9 μM, respectively. The NAD(P)H-dependent kinetics with glyoxylate for the two OsGRs were also determined. The NADPH-linked *K*_*m*(glyoxylate)_ values for OsGR1 and OsGR2 were 30.4 and 72.1 μM, and the NADH-linked *K*_*m*(glyoxylate)_ values were 267.7 and 144.6 μM (Table 3), respectively. Besides, the *V*_*max*_ of OsGR1 with NAD(P) H were higher than that of OsGR2. It is noteworthy that glyoxylate is an efficient precursor for oxalate biosynthesis in plants, and rice leaves contain high levels of oxalate ranging from 25 to 120 mM [14, 17, 25]. Therefore, we tested if oxalate had a feedback effect on the glyoxylate reduction catalyzed by OsGRs. As shown in Table 4, when using NADPH as cofactor, the *K*_*i*_ value of oxalate for OsGR1 was 21.2 mM and that for OsGR2 was 290.8 mM. When using NADH as cofactor, the *K*_*i*_ values of oxalate were much lower, 3.6 mM for OsGR1, and 8.2 mM for OsGR2. The above results indicated that OsGR1 and OsGR2 have high affinity for glyoxylate, and both prefer NADPH to NADH as cofactor, with the NADH-dependent activity much more sensitive to oxalate inhibition than the NADPH-dependent activity.

**Table 3:** *K*_*m*_ and *V*_*max*_ of OsGR1 and OsGR2 with various substrates

Enzyme	Substrate at fixed concentration	Substrate at varying concentration	*K*_*m*_ (μM)	*V*_*max*_ (μmol glyoxylate min^−1^ mg^−1^ protein)
OsGR1	Glyoxylate	NADPH	17.6 ± 3.1	74.7 ± 5.9
	NADPH	Glyoxylate	30.4 ± 3.5	
	Glyoxylate	NADH	420.2 ± 50.5	59.9 ± 8.1
	NADH	Glyoxylate	267.7 ± 22.7	
OsGR2	Glyoxylate	NADPH	53.0 ± 4.6	61.9 ± 3.2
	NADPH	Glyoxylate	72.1 ± 5.3	
	Glyoxylate	NADH	403.9 ± 66.5	22.4 ± 3.5
	NADH	Glyoxylate	144.6 ± 19.3	

The OsGR1 and OsGR2 expressed in *E.coli* were purified for analyses, and *K*_*m*_ values with different substrate pairs were compared. Values are means ± SD (*n = 3*)

**Table 4:** *K*_*i*_ of OsGR1 and OsGR2 with oxalate

Enzyme	*K*_*i*(oxalate)_ (mM)	
	Glyoxylate as substrate NADPH as cofactor	Glyoxylate as substrate NADH as cofactor
OsGR1	21.2 ± 4.3	3.6 ± 0.8
OsGR2	290.8 ± 59.7	8.2 ± 1.1

Values are means ± SD (*n = 3*)

### Characterization of *OsGR*-genetically modified plants

**Fig. 3:**
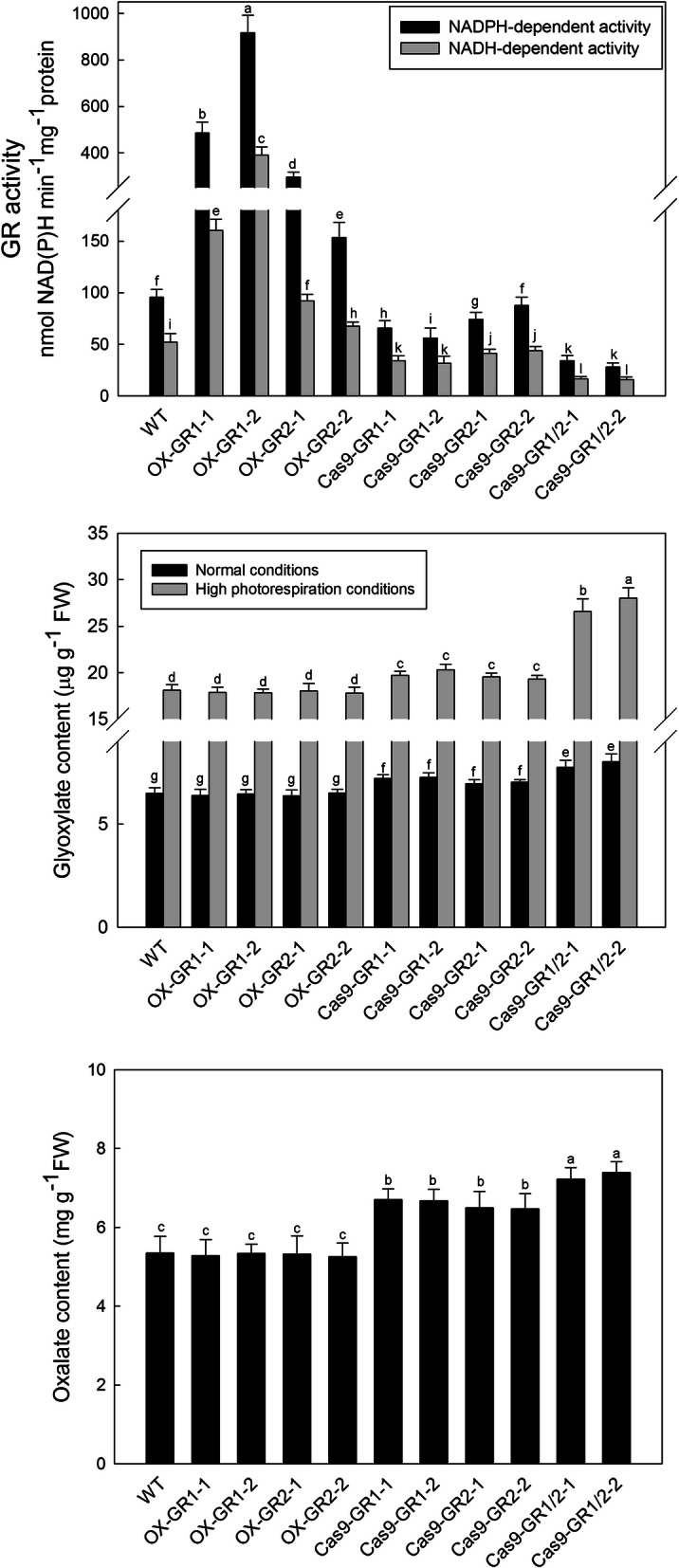
Catalytic characteristics of OsGR isoforms in vivo. **a** NAD(P)H-dependent glyoxylate reducing activities in different *OsGR*-genetically modified plants. **b** The accumulation of glyoxylate in different *OsGR*-genetically modified plants under normal growth conditions and high photorespiration conditions. **c** The accumulation of oxalate in different *OsGR*-genetically modified plants under normal growth conditions. OX-GR1 and OX-GR2 represent the *OsGR1* and *OsGR2* overexpression plants; Cas9-GR1 and Cas9-GR2 represent the *OsGR1* and *OsGR2* single knockout mutants; Cas9-GR1/2 represent the *OsGR1* and *OsGR2* double knockout mutants. Values are means ± SD of three replicates. Means denoted by the same letter did not significantly differ at *P* < 0.05 according to Duncan’s multiple range tests

Thus far, few data could be found on the biological roles of OsGR isoforms, particularly for rice. In this study, the open reading frames of *OsGR1* and *OsGR2* were used to construct the *OsGR*-overexpression transgenic lines, the knockout lines were generated by CRISPR-Cas9 system, and the T2 heterozygous lines were used for the analysis here. As expected, in leaves of *OsGR*-overexpression (OX*-OsGR*) lines each *OsGR* gene was up-regulated at mRNA levels (Additional file 2), and correspondingly, the GR activities were elevated by 5–8-folds in OX*-OsGR1* lines and 1–3-folds in OX*-OsGR2* lines (Fig. 3a). When *OsGR1* and *OsGR2* were knocked out using pYLCRISPR/Cas9 system (Additional files 2 and 3), GR activities were decreased by 35–45% and 20–25% in leaves of *OsGR1* and *OsGR2* knockout mutants, respectively, indicating that OsGR1 is the major contributor to total GR activities (Fig. 3a). In the *OsGR1*/*OsGR2* double mutant, the GR activities dropped by 60–75% (Fig. 3a). The residual activities might come from some other enzymes that were reported to reduce glyoxylate as a side reaction, such as hydroxypyruvate reductases (HPR) [5, 26]. Subsequently, glyoxylate levels in these genetically modified rice lines were analysed. Under normal growth conditions, the glyoxylate content of WT was about 6.5 μg g^− 1^ FW, and increased by 0.4–0.8 μg g^− 1^ FW in *OsGR1* and *OsGR2* knockout mutants, and 1.2–1.5 μg g^− 1^ FW in *OsGR1/OsGR2* double mutant, while no obvious decreases in glyoxylate contents were observed in OX-*OsGR* lines (Fig. 3b). Moreover, all plants accumulated much more glyoxylate under high photorespiration conditions, with WT being increased to approximately 18.0 μg g^− 1^ FW, and *OsGR1/OsGR2* double mutant up to 28.0 μg g^− 1^ FW (Fig. 3b). In addition, oxalate levels of these rice lines under normal growth conditions were measured. The oxalate contents increased ranging from 20 to 25% in the *OsGR1* and *OsGR2* single knockout mutants, and from 30 to 40% in the *OsGR1/OsGR2* double mutants (Fig. 3c). No changes were observed in both OX-*OsGR1* and OX-*OsGR2* lines (Fig. 3c).

**Fig. 4:**
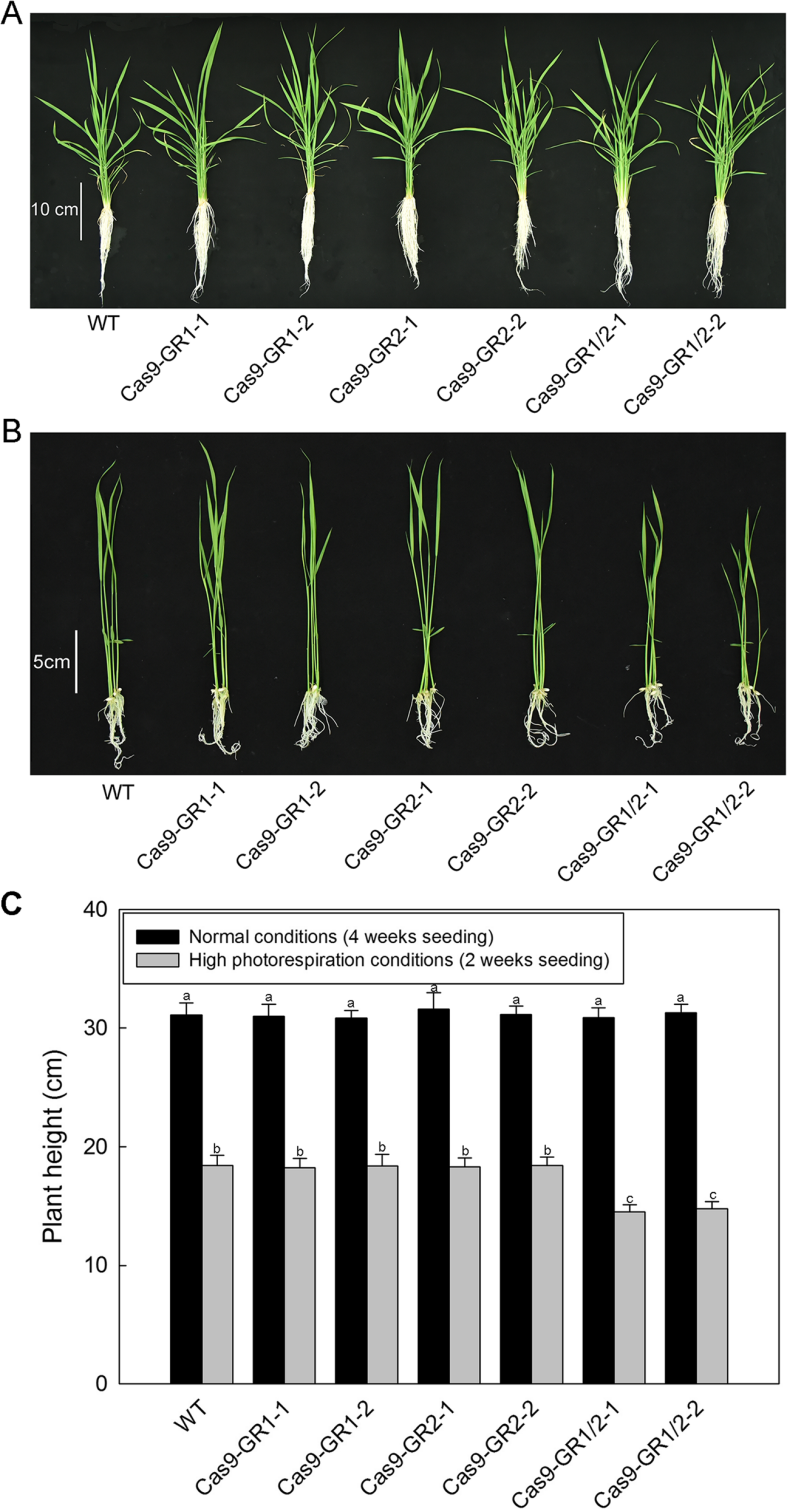
Growth phenotypes of the *OsGR*-knockout mutants. **a** Phenotypes of the *OsGR*-knockout mutants growth under normal condition (4 weeks seedling). Scale bar, 10 cm. **b** Phenotypes of the *OsGR*-knockout mutants growth under photorespiration-promoted conditions. Scale bar, 5 cm (2 weeks seedling). **c** Plant height. Cas9-GR1 and Cas9-GR2 represent the *OsGR1* and *OsGR2* single knockout mutants; Cas9-GR1/2 represent the *OsGR1* and *OsGR2* double knockout mutants. Values are means ± SD of three replicates. Means denoted by the same letter did not significantly differ at *P* < 0.05 according to Duncan’s multiple range tests

Under normal conditions, these *OsGR*-genetically modified plants showed no significant phenotypic differences as compared with WT (Fig. 4a). However, when the photorespiration was promoted by low CO_2_ concentration and continuous light [27, 28], the *OsGR1/OsGR2* double mutants showed stunted growth (Fig. 4b, Additional file 4). The OX-*OsGR1* and OX-*OsGR2* lines displayed no apparent phenotypic differences under both normal and photorespiration-promoted conditions (data not shown). These results stronly support that OsGR1 and OsGR2 are involved in glyoxylate detoxification under certian stressful conditions which stimulate photorespiration, although both may function redundantly at most of the time.

## Discussion

Biological roles of different gene members in multigene family could be diverse in general [29–33]. The *GR* multigene family exists in various plant species, such as *Arabidopsis*, *Sinorhizobium meliloti*, apple and rice [2, 7], and their GR isoforms display different expression patterns, biochemical features and functions. It has been reported that GR might be involved in the detoxification of glyoxylate and succinic semialdehyde in *Arabidopsis* and tobacco under certain stressfull conditions [1, 6]. Zarei et al. [7] further demonstrated that the NADPH/NADH ratio might regulate the expression of GR in *Arabidopsis* and mediate their roles in the aldehydes detoxification. However, by reviewing the literature, little detailed information is available about the physiological roles of GR in rice.

Here, we first show that the NADPH-dependent glyoxylate reducing activity is the major GR activity in different tissues and at different growth stages of rice (Fig. 1a, b), similar to the GR from spinach and tobacco [4, 34]. Further, analyses at transcription levels and protein abundance suggest that OsGR1 is the major GR isoform in rice (Fig. 1c, Table 2). Subcellular localization of GR remains controversial, and recent research demonstrated that the *Arabidopsis* AtGR1 was localized in cytoplasm, while AtGR2 was localized in plastids and mitochondria [7, 20]. In the present study, confocal microscopy observation localized OsGR1 in the cytoplasm, while OsGR2 in the chloroplast (Fig. 1d), and western blot assay further proved that OsGR2 is a chloroplastic protein (Fig. 1e).

Determing enzymatic properties is a conventional way to indirectly reveal biological functions of enzymes [29, 35, 36]. GR proteins were first purified from tobacco and spinach leaves and then biochemically and enzymatically characterized, showing that the glyoxylate-linked *K*_*m*(NADPH)_ values of GR from tobacco and spinach are 0.32 and 0.13 mM, respectively [4]. GR isoforms from *Arabidopsis*, apple and rice showed catalytic activities not only to reduce glyoxylate but also to reduce succinic semialdehyde [7]. In this study, glyoxylate-dependent enzymatic properties of OsGR1 and OsGR2 were comparatively studied. OsGR1 and OsGR2 showed the highest activities in the physiological pH range (7.0–7.5) (Fig. 2b), and both prefer NADPH to NADH as cofactor (Table 3), consistent with GR isozymes from other species [2, 4, 6]. As shown in Table 3, the NADPH-linked *K*_*m*(glyoxylate)_ of OsGR1 was 30.2 μM, significantly lower than that of OsGR2 (72.1 μM), indicating that OsGR1 has a higher affinity for glyoxylate than OsGR2. Nevertheless, the physiological concentrations of glyoxylate in plants range from 100 to 300 μM [17, 18, 37], which are higher than the NADPH-linked *K*_*m*(glyoxylate)_ values of OsGR1 and OsGR2. Therefore, both OsGR1 and OsGR2 may efficiently catalyze the reduction of glyoxylate at physiological concentrations in rice. In addition, rice is an oxalate-accumulating plant, oxalate contents in rice range from 20 to 100 mM [14, 25]. Based on the *K*_*i*(oxalate)_ of OsGR1 and OsGR2 (Table 4), under physiological conditions, the endogenous oxalate might be able to partially suppress the NADPH-dependent glyoxylate reducing activity of OsGR1, but not OsGR2, as a result, it is likely that in vivo OsGR1 and OsGR2 may work in a similar efficient manner to reduce glyoxylate. However, the NADH-dependent glyoxylate reducing activity of OsGR1 and OsGR2 could be inhibited by the physiological concentrations of oxalate, such that the NADH-dependent activities of GR may have not actually functioned in the glyoxylate detoxification in rice plants.

Photorespiratory glyoxylate is produced in peroxisomes and usually accumulated under high photorespiration or other stressful conditions, which is highly toxic to plant cells if not metabolized or removed timely [38, 39]. Moreover, glyoxylate is a small organic acid, which can diffuse from peroxisomes to other cellular compartments [11, 19, 40–44]. Previous studies assumed that photorespiratory glyoxylate escaping from peroxisomes could be scavenged by GRs, but direct molecular genetic evidences are still scarce [10, 11, 13]. In order to further verify if OsGR1 and OsGR2 function in the glyoxylate detoxification in vivo, various *OsGR*-genetically modified rice lines were generated and the glyoxylate contents were detected in those transgenic plants. It was noticed that the WT and OX-OsGR lines had same levels of glyoxylate even under high photorespiration conditions (Fig. 3b), likely because the background GR activities in WT are high enough to remove the photorespiratory glyoxylate leaking from peroxisome, so that further overexpression would be needless. Previous studies have also reported that there are certain anaplerotic reactions that compensate for the loss of glyoxylate in plants [14, 18, 19, 38]. Nevertheless, the glyoxylate levels were increased in the *OsGR* knockout mutants, particularly in the double mutants (Fig. 3b), and this value became much higher under high photorespiration conditions (Fig. 3b). Unexpectedly, however, neither their single nor the double mutants showed phenotypic changes under normal conditions (Fig. 4a, c), negative phenotypes could be noticed for the double mutant only under high photorespiration conditions (Fig. 4b, c and Additional file 4). This implicates that the glyoxylate accumulation due to deficiency of OsGR1 and OsGR2 might have not yet attained the toxic threshold for rice plants under normal conditions, and the two OsGR isoforms are both required for efficiently removing glyoxylate under high photorespiration conditions (e.g. drought, heat, high light). Overall, the datas stronly support that OsGR1 and OsGR2 are indeed involved in glyoxylate detoxification under certian stressfull conditions that stimulate photorespiration, although both may function redundantly sometimes. In addition, oxalate contents of various *GR*-genetically modified rice lines were also analyzed. As seen from the Fig. 3c, along with the glyoxylate increase of *OsGR* knockout mutants (Fig. 3b), oxalate was increased by 25–30% in both the *OsGR1* and *OsGR2* single mutants, and by 30–40% in the *OsGR1*/*OsGR2* double mutants. These results further confirmed our previous conclusion that glyoxylate is an efficient precursor for oxalate biosynthesis, and both OsGR1 and OsGR2 may be involved in the regulation of oxalate metabolism in rice. Molecular mechanisms of these biological processes remain obscure and await further investigation.

## Conclusions

GR activities not only show interdiurnal variations, but also fluctuate during different growth stages. OsGR1 is a cytoplasmic protein, while OsGR2 is localized in the chloroplast. OsGR1 contributes more to total GR activities, and has a higher affinity for glyoxylate than OsGR2, although both can efficiently catalyze the reduction of glyoxylate using NADPH as preferred cofactor in rice plants. Under normal conditions, OsGR1 and OsGR2 are involved in the detoxification of glyoxylate in a nonobligatory manner, likely because the accumulated glyoxylate due to deficiency of OsGR1 and OsGR2 may have not attained the toxic threshold for rice plants under normal conditions. Nevertheless, the two OsGR isoforms are simultaneously required to cope with the over-production of glyoxylate under high photorespiration conditions.

## Methods

### Plant materials and growth conditions

*Oryza sativa* cv. Zhonghua 11 (japonica cultivar-group) preserved by our laboratory was used for generation of the transgenic lines and the functional analyses (Xu et al., 2006). Pre-germinated rice seeds were normally grown in KimuraB complete nutrient solution [45] under greenhouse condition (14 h light/10 h dark, average temperature of 30/25 °C (light/dark), relative humidity 60–80% and average light intensity of 600 μmol m^− 2^ s^− 1^). In order to promote photorespiration, the rice plants were cultivated in enclosed glass tubes (21 cm height, 3.0 cm diameter) under continuous light, and then the CO_2_ levels in the tubes decreased rapidly, facilitating the flux of the photorespiration [27, 28].

### Plasmid construction

Total RNA was extracted from rice leaves using EZ-10 Total RNA Mini-Preps Kit (Sangon Biotech, China), and the purified RNA was assessed with a NanoPhotometer (IMPLEN, Germany). First-strand cDNA was synthesized using HiScript 1st Strand cDNA Synthesis Kit (Vazyme, China). Primers were designed to include the complete open reading frames of two GR genes. To generate GR-overexpression transgenic lines, each GR sequence was cloned into pYLox.5 vector using ClonExpress Ultra One Step Cloning Kit (Vazyme, China). For the generation of CRISPR-Cas9 knockout lines, specific targeting sequences of *OsGR1* and *OsGR2* (Additional file 3) were synthesized and cloned into pYLCRISPR/Cas9Pubi vector [46]. To construct the vectors for protein heterologous expression in *E.coli*, the full-length *OsGR1* and the truncated *OsGR2* (without N-terminal CTP) sequences were cloned into pColdI vector. The sequence of the first 879 bp of the *OsGR1* and the first 153 bp of the *OsGR2* were used to generate the *OsGR1*-GFP-pBI121 and *OsGR2*-GFP-pBI121vectors [33].

### RNA isolation and qRT-PCR

First-strand cDNA was synthesized using HiScript 1st Strand cDNA Synthesis Kit as above (Vazyme, China). The specific primer pairs were designed for the qRT-PCR of *OsGR1* and *OsGR2* (Additional file 5). The qRT-PCR reaction mixture consisted of 0.2 μM (each) primer, 10 μL of 2 × SYBR Green PCR Master Mix (Toyobo, Japan), and 2 μL of appropriate diluted cDNA. The analysis was conducted using a DNA Engine Opticon 2 Real-Time PCR Detection system and Opticon Monitor software (Bio-Rad, CA). The data were normalized to the amplification of the *OsActin1* gene (Os03g0718100).

### Quantitative proteomic analysis

Protein extracts were obtained according to Lu et al. (2013) with some modifications [38]. Crude protein extracts were obtained by grinding 1.0 g rice leaves in liquid nitrogen followed by resuspension in 5 ml ice-cold Tris-HCl (100 mM, pH 8.5) containing 6 M guanidine chloride (GdnHCl) and 1% protease inhibitor cocktail (Abcam, USA), protein amount was quantified by BCA assay (Thermo Fisher Scientific, USA). Subsequently, 200 μg of lysate was added to four volumes of methanol followed by an equal volume of chloroform with mixing, three volumes of ddH_2_O were added to the tube with mixing. The solution was centrifuged at 14,000 g for 5 min. The upper aqueous layer was discarded, the protein pellet was washed with four volumes of methanol, and the tube was centrifuged again. The supernatant was discarded, and the precipitated protein pellet was air-dried. Afterthat, the chromatography-tandem mass spectrometry (LC-MS/MS) analysis was performed by BGI (BGI-Shenzhen, China).

### Generation of genetically modified rice lines

The various constructed vectors were transformed into rice callus by *Agobacterium*-mediated infection (strain *EHA105*) [47]. The T1 seeds from the positive T0 lines were germinated in complete Kimura B nutrient solution, and then transplanted to soil to grow until the T2 seeds were harvested. Afterthat, The T2 heterozygous plants that originated from two independent lines were used for the determination of GR activity and stress treatments.

### Expression of proteins in *E. coli* and their purification

The OsGR sequences were cloned into pColdI vector, transformed into *E. coli* Rosseta (DE3) cells and screened on LB-plates containing ampicillin. The proteins were inductively expressed in *E. coli* as previously described with some modifications [48]. Briefly, the transformed *E. coli* cells were incubated with 1 mM of isopropyl-thio-β-D-1-thiogalactopyranoside (IPTG) at 16 °C for 10–16 h with shaking at 180 rpm. After that, the supernatant of the *E. coli* cell lysate was purified according to the manufacturer’s protocol of Ni-IDA resin (Bio-Rad, USA). The Ni-IDA resin (Bio-Rad) was packed in a Bio-Scale MT5 column (10 × 64 mm) up to 2 mL, and then equilibrated with 3 column volumes (CV) of wash buffer (50 mM PBS, pH 8.0, 10 mM imidazole, 300 mM NaCl). The filtered supernatant was prepared by mixed with an equal volume of binding buffer (100 mM PBS, pH 8.0, 20 mM imidazole, 600 mM NaCl), and loaded onto the Ni-IDA resin column. The column was washed with 10 CV of wash buffer, and the bound proteins were eluted with 5 CV of 50 mM PBS (pH 8.0) containing 150 mM imidazole and 300 mM NaCl. The eluted fractions were desalted by ultrafiltration and checked by 12% SDS-PAGE. All the purified proteins were stored in 50 mM PBS (pH 7.0) containing 10% glycerol at − 75 °C for subsequent assay.

### GR activity assay

The GR catalytic activity was monitored as the oxidation of NAD(P) H at physiological temperature (30 °C) according to Zarei et al. [7], unless specific variations were needed in different kinetic and characterization investigations. A typical reaction mixture contains 50 mM PBS (pH 7.4), 0.2 mM NADPH (or 1.0 mM NADH) and 1 mM glyoxylate. The effects of varying pH were assayed with 50 mM PBS from pH 5.8 to 8.5. For kinetic parameter determination, catalyses of substrates were performed over a range of concentration (0.01–0.1 mM NADPH for determining *K*_*m*(NADPH)_; 0.1–1.0 mM NADH for determining *K*_*m*(NADH)_; 0.01–0.1 mM glyoxylate for determining *K*_*m*(glyoxylate)_ with NADPH as cofactor; 0.1–0.5 mM glyoxylate for determining *K*_*m*(glyoxylate)_ with NADH as cofactor), the *K*_*m*_ was calculated from double-reciprocal plots according to the method of Lineweaver and Burk. Moreover, slopes of the reciprocal plots were then plotted against the concentration of oxalate (10–300 mM with NADPH as cofactor, 2.0–15.0 mM with NADH as cofactor) to evaluate *K*_*i*_ data [49].

### Subcellular localization and western blot analysis

The rice protoplasts were isolated according to Zhang et al. [50], approximate 10 μg of GFP-tagged constructs were transfected into 100 μL of protoplasts (about 2.0 × 10^5^ cells) by PEG-mediated transfection, and then the protoplasts were incubated in the dark at 25 °C for 14–16 h [50, 51]. The confocal images were captured by a LSCM 780 system (Zeiss, Germany). Proteins extracted from rice protoplasts expressed OsGR2-GFP were separated on 12% SDS polyacrylamide gel (SDS-PAGE) and electro-blotted onto a nitrocellulose membrane using wet transfer, and the OsGR2-GFP was detected using a GFP-antibody (Abcam, USA).

### Determination of glyoxylate and oxalate

Glyoxylate and oxalate were determined as reported by Xu et al. [17]. Aliquots of 0.2–0.5 g leaves, depending on sample availability, were homogenized in 1–4 mL of 0.5 N HCl and heated at 80 °C for 10 min. After that, the homogenate was added distilled water up to 5–25 mL, and 2–3 mL of the solution was centrifuged at 12000 rpm for 10 min and passed through a filter (0.45 μm) before HPLC analysis.

Glyoxylate could react with phenylhydrazine to form phenylhydrazone, and then the derivative was quantified by HPLC. Hypsil C18 column (5 μm, 4.6 × 250 mm) equipped Waters 550 (Waters, USA) was used as the static phase, a mobile phase containing 5% methanol and 95% phosphate buffer (13 mM potassium biphosphate; 1 mM potassium phosphate dibasic pH 6.0) and detection at 324 nm were applied in this system. For determination of oxalate, the mobile phase was a solution containing 0.5% KH_2_PO_4_ and 0.5 mM tetrabutylammonium hydrogen sulphate (TBA) buffered at pH 2.0 with orthophosphoric acid, flow rate was 1 mL min^− 1^ and detected at 220 nm.

## Supplementary information

**Additional file 1.** Multiple sequence alignment (MSA) of OsGR and AtGR isoforms at the level of protein and nucleotide.

**Additional file 2. ***OsGR1* and *OsGR2* transcript abundances in the leaves of different *OsGR*-genetically modified plants were determined by qRT-PCR. Relative mRNA levels in various rice lines were graphed based on the *OsGR1* mRNA level in WT as 1.

**Additional file 3. **Molecular evalution of the Crispr-Cas9 generated *OsGR1* and *OsGR2* single and double mutants.

**Additional file 4. ***OsGR*-knockout mutants growth under photorespiration-promoted conditions. Cas9-GR1 and Cas9-GR2 represent the *OsGR1* and *OsGR2* single knockout mutants; Cas9-GR1/2 represent the *OsGR1* and *OsGR2* double knockout mutants. These results are representative of three independent experiments.

**Additional file 5.** The primers used for real-time quantitative PCR.

## Data Availability

All data generated or analysed during this study are included in this published article and its supplementary information files.

## Abbreviations

GR: Glyoxylate reductase
SSA: Succinic semialdehyde
CTP: Chloroplast transit peptides
qRT-PCR: Real-time quantitative PCR
GFP: Green fluorescence protein
TBA: Tetrabutylammonium hydrogen sulphate
IPTG: Isopropyl-thio-β-D-1-thiogalactopyranoside
