# Supplementary material for: Two glyoxylate reductase isoforms are functionally redundant but required under high photorespiration conditions in rice
Source: BMC Plant Biol. 2020 Jul 29;20:357. doi: 10.1186/s12870-020-02568-0 (PMC7391683; doi:10.1186/s12870-020-02568-0)
Supplement: Supplementary file 1 — Additional file 1. Multiple sequence alignment (MSA) of OsGR and AtGR isoforms at the level of protein and nucleotide. [file 12870_2020_2568_MOESM1_ESM.docx]

**Additional file 1** Multiple sequence alignment (MSA) of OsGR and AtGR isoforms

at the level of protein and nucleotide.

**MSA at the protein level.**

**MSA at the nucleotide level.**
