# Supplementary material for: Two glyoxylate reductase isoforms are functionally redundant but required under high photorespiration conditions in rice
Source: BMC Plant Biol. 2020 Jul 29;20:357. doi: 10.1186/s12870-020-02568-0 (PMC7391683; doi:10.1186/s12870-020-02568-0)
Supplement: Supplementary file 2 — Additional file 2. OsGR1 and OsGR2 transcript abundances in the leaves of different OsGR-genetically modified plants were determined by qRT-PCR. Relative mRNA levels in various rice lines were graphed based on the OsGR1 mRNA level in WT as 1. [file 12870_2020_2568_MOESM2_ESM.docx]

**Additional file 2** *OsGR1* and *OsGR2* transcript abundances in the leaves of different *OsGR*-genetically modified plants were determined by qRT-PCR. Relative mRNA levels in various rice lines were graphed based on the *OsGR1* mRNA level in WT as 1.

**
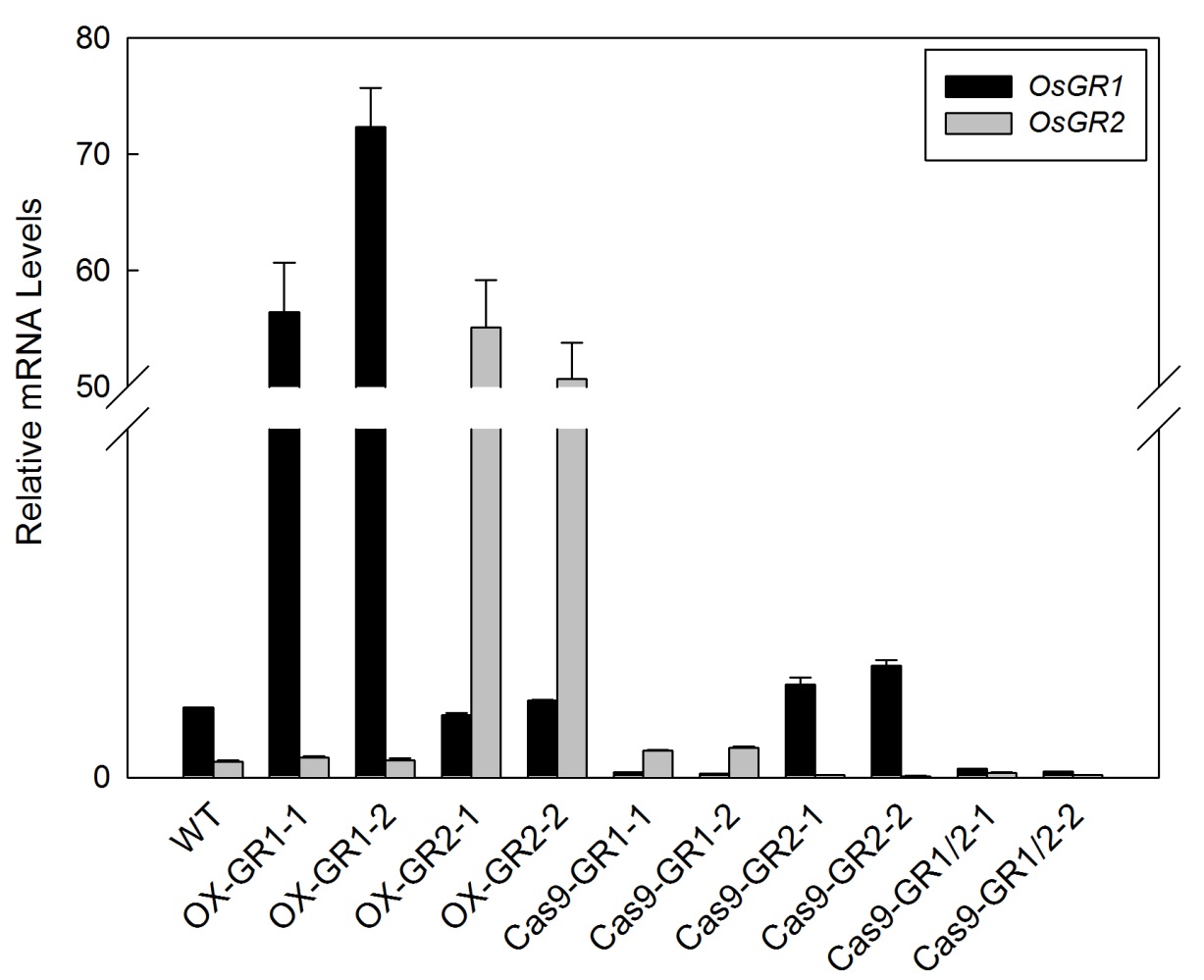
**
