# Supplementary material for: Two glyoxylate reductase isoforms are functionally redundant but required under high photorespiration conditions in rice
Source: BMC Plant Biol. 2020 Jul 29;20:357. doi: 10.1186/s12870-020-02568-0 (PMC7391683; doi:10.1186/s12870-020-02568-0)
Supplement: Supplementary file 3 — Additional file 3. Molecular evalution of the Crispr-Cas9 generated OsGR1 and OsGR2 single and double mutants. [file 12870_2020_2568_MOESM3_ESM.docx]

**Additional file 3** Molecular evalution of the Crispr-Cas9 generated *OsGR1* and *OsGR2* single and double mutants.

**Cas9-*OsGR1***

WT: TGTTTGAGTGCGCAGTGCCAGG

Cas9-*OsGR1-1*: TGTTTGAGTGCGCAGT**T**GCCAGG

Cas9-*OsGR1-2*: TGTTTGAGTG-------GCCAGG

**Cas9-*OsGR2***

WT: TACAGGTACGAGCCTTCACCGG

Cas9-*OsGR1-1*: TACAGGTACGAGCCTT**T**CACCGG

Cas9-*OsGR1-2*: TACAGGTACGAG----CACCGG

**Cas9-*OsGR1/2***

WT: GTGTTTGAGTGCGCAGTGCCAGG (*OsGR1*)

CTACAGGTACGAGCCTTCACCGG (*OsGR2*)

Cas9-*OsGR1/2-1*: GTGTTTGA-----CAG---AGG (*OsGR1*) CTACAGGTACGAGCCT-CACCGG (*OsGR2*)

Cas9-*OsGR1/2-2*: GTGTTTGAGTGC----TGCCAGG (*OsGR1*)

CTACAGGTACGAGCC—CACCGG (*OsGR2*)
