# Supplementary material for: Two glyoxylate reductase isoforms are functionally redundant but required under high photorespiration conditions in rice
Source: BMC Plant Biol. 2020 Jul 29;20:357. doi: 10.1186/s12870-020-02568-0 (PMC7391683; doi:10.1186/s12870-020-02568-0)

**Additional file 4** *OsGR*-knockout mutants growth under photorespiration-promoted conditions. Cas9-GR1 and Cas9-GR2 represent the *OsGR1* and *OsGR2* single knockout mutants; Cas9-GR1/2 represent the *OsGR1* and *OsGR2* double knockout mutants. These results are representative of three independent experiments.


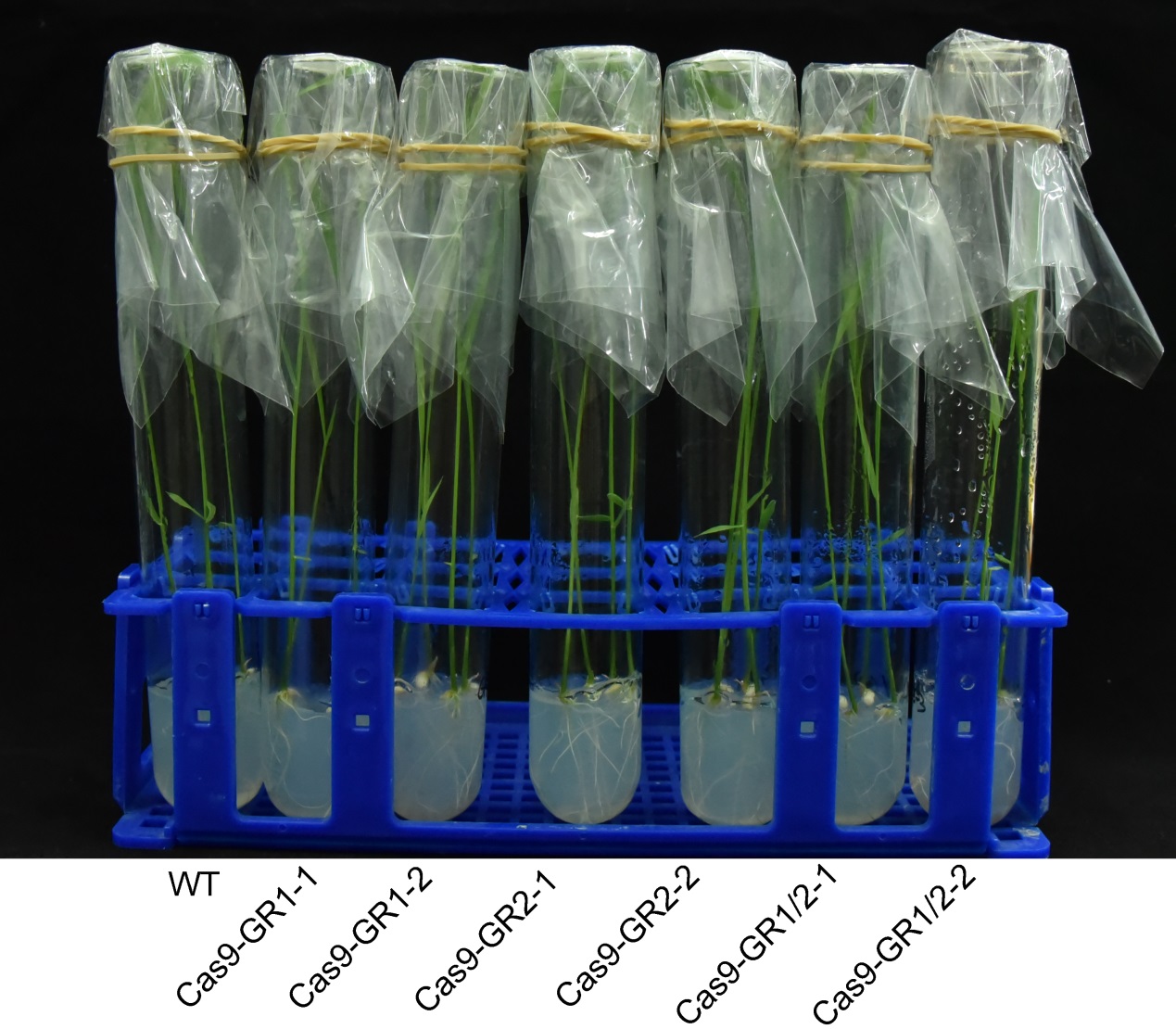

Supplement: Supplementary file 4 — Additional file 4. OsGR-knockout mutants growth under photorespiration-promoted conditions. Cas9-GR1 and Cas9-GR2 represent the OsGR1 and OsGR2 single knockout mutants; Cas9-GR1/2 represent the OsGR1 and OsGR2 double knockout mutants. These results are representative of three independent experiments. [file 12870_2020_2568_MOESM4_ESM.docx]
