# Supplementary material for: Two glyoxylate reductase isoforms are functionally redundant but required under high photorespiration conditions in rice
Source: BMC Plant Biol. 2020 Jul 29;20:357. doi: 10.1186/s12870-020-02568-0 (PMC7391683; doi:10.1186/s12870-020-02568-0)
Supplement: Supplementary file 5 — Additional file 5. The primers used for real-time quantitative PCR. [file 12870_2020_2568_MOESM5_ESM.docx]

**Additional file 5** The primers used for real-time quantitative PCR.

| Gene | Primer sequences |
| --- | --- |
| *OsGR1* | *OsGR1*-qRT-F: 5-GCGAATCCAATGTTCAAGC-3  *OsGR1*-qRT-R: 5-CGTAAACCGCTGAGAAATCC-3 |
| *OsGR2* | *OsGR2*-qRT-F: 5-AGGGTTCAGTGGGAAGGTG-3  *OsGR2*-qRT-R: 5-AAGGTCACATCGCAAGACG-3 |
| *OsActin1* | *OsActin1*-qRT-F: 5-CTTCATAGGAATGGAAGCTGCG-3  *OsActin1*-qRT-R: 5-CACCTTGATCTTCATGCTGCTA-3 |
